# Prevalence and risk factors of childbirth-related post-traumatic stress symptoms

**DOI:** 10.1186/1471-2393-12-88

**Published:** 2012-09-03

**Authors:** Maryam Modarres, Sedigheh Afrasiabi, Parvin Rahnama, Ali Montazeri

**Affiliations:** 1 Department of Midwifery, Faculty of Nursing and Midwifery, Tehran University of Medical Sciences, Tehran, Iran; 2 Department of Midwifery, Faculty of Nursing and Midwifery, Bushehr University of Medical Sciences, Bushehr, Iran; 3 Department of Midwifery, Faculty of Nursing and Midwifery, Shahed University, Tehran, Iran; 4 Mental Health Research Group, Health Metrics Research Centre, Iranian Institute for Health Sciences Research, ACECR, Tehran, Iran

## Abstract

**Background:**

There is evidence that traumatic birth experiences are associated with psychological impairments. This study aimed to estimate the prevalence of childbirth-related post-traumatic stress symptoms and its obstetric and perinatal risk factors among a sample of Iranian women.

**Methods:**

This was a cross-sectional study carried out in Bushehr, Iran during a 3-months period from July to September 2009. Data were collected from all women attending eleven healthcare centers for postnatal care 6 to 8 weeks after childbirth. Those who had a traumatic delivery were identified and entered into the study. In order to assess childbirth-related post-traumatic stress, the Post-traumatic Symptom Scale-Interview (PSS-I) was administered. Data on demographic, obstetric and perinatal characteristics also were collected. Multivariate logistic regression was performed to examine the association between childbirth-related post-traumatic stress and demographic and obstetric and perinatal variables.

**Results:**

In all, 400 women were initially evaluated. Of these, 218 women (54.5%) had a traumatic delivery and overall, 80 women (20%) were found to be suffering from post-partum post-traumatic stress disorder (PTSD). Multiple logistic regression analysis revealed that post-partum PTSD was associated with educational level, gestational age at delivery, number of prenatal care visits, pregnancy complications, pregnancy intervals, labor duration, and mode of delivery.

**Conclusions:**

The findings indicated that the prevalence of traumatic birth experiences and post-partum PTSD were relatively high among Iranian women. The findings also indicated that obstetric and perinatal variables were independently the most significant contributing factors to women’s post-partum PTSD. It seems that a better perinatal care and supportive childbirth might help to reduce the burden of post-partum PTSD among this population.

## Background

The post-traumatic stress disorder (PTSD) after childbirth was initially described by Bydlowski and Raoul-Duval [1]. Several studies indicated that childbirth is associated with post-traumatic stress disorder [2-5] and it can disrupt childcare in infancy [6]. In addition it has been shown that pregnancy occurrence after a traumatic birth experience may also result in the seek abortion, or request for unnecessary cesarean section in subsequent pregnancies [7,8].

The PTSD is an anxiety disorder that can occur secondary to exposure to extreme events (stressors) that are at outside of the usual range of human experience [9]. Childbirth is a physiologic and predictable event. Thus, the American Physiatrist Association was unable to classify childbirth as a traumatic disorder. However, since the Diagnostic and Statistical Manual of Mental Disorders-Revision 4 (DSM-IV) re-defined the criterion for a stressor, it was suggested that this new definition might also apply to childbirth as a traumatic event [9].

There is evidence that traumatic birth experiences are associated with post-traumatic stress disorder symptoms [10-12]. Studies have shown that different risk factors contribute to the development of post-partum PTSD including history of psychological problems, trait anxiety, obstetric procedures, negative aspects in staff-mother contact, sense of control, and social support [5,13]. Even, studies indicated that a history of trauma, in particular childhood sexual abuse (CSA), might be a risk factor for higher distress levels in pregnancy, and for post-partum post-traumatic stress symptoms [14,15]. In addition several studies revealed that the obstetrics factors and complicated deliveries were the most significant indicators of the post-partum PTSD [16-18]. However, if risk factors for PTSD were identified in pregnancy, primary prevention might be possible [2], and it could be prevented by support from health care providers.

Every year about 1,363,542 women in Iran give birth [19]. Of these it is estimated that 35% of deliveries are cesarean sections and very likely subject to a traumatic birth [20]. There are limited reports about the post-partum PTSD in Iran [21,22]. Thus, the aim of present study was to estimate the prevalence of childbirth-related post-traumatic stress symptoms and its obstetric and perinatal risk factors among a sample of Iranian women. By examining the risk factors associated with PTSD after childbirth health practitioners are better able to help with prevention or management of possible posttraumatic stress symptoms associated with childbirth.

## Methods

### Design and data collection

This was a cross sectional study carried out in Bushehr, Iran during a 3-months period from July to September 2009. Data were collected from all women attending eleven healthcare centers for postnatal care 6 to 8 weeks after childbirth. They were interviewed to indicate those who experienced a traumatic birth. One of us (SA) carried out all interviews in a private setting. Women who did not experience a traumatic delivery and those with stillbirth or neonatal deaths were excluded. Subsequently, the following questionnaires were completed for women who experienced a traumatic childbirth.

### Questionnaires

1. A study specific questionnaire in order to collect data on demographic, obstetric and perinatal information.

2. The Post-traumatic Symptom Scale-Interview (PSS-I): This is a 17-items questionnaire that assesses PTSD symptoms based on the Diagnostic and Statistical Manual of Mental Disorders-Revision 4 (DSM-IV) [9]. It contains three subscales namely: re-experiencing, avoidance and increased arousal symptoms. Items are rated on 0–3 scales for combined frequency and severity, yielding one score per item. A score of 0 corresponds to ‘not at all’; 1 corresponds to ‘once per week or less/a little’; 2 corresponds to ‘2 to 4 times per week/somewhat’; corresponds to ‘5 or more times per week/very much’. Severity scores for each of the re-experiencing, avoidance, and increased arousal subscales could be obtained by summing scores for items within each subscale. PTSD severity score could be obtained by summing all 17 items [23,24]. To make a diagnosis of post-partum PTSD, at least one re-experiencing item, three avoidance items and two increased arousal items should be present [9]. Validity of the Iranian version of PSS-I for PTSD screening is well documented [25].

### Analysis

Participants were classified as with and without post-partum PTSD based on their PSS-I diagnosis. Descriptive statistics including chi-square were used to explore the data. Forward conditional multiple logistic regression analysis was performed to examine the association between dependent (post-partum PTSD) and independent variables. The level of significance was set at 5%. The SPSS version 16 was used to analyze the data.

### Ethics

The ethics committee of Tehran University of Medical Sciences approved the study. We obtained written informed consent from participants after comprehensive explanation of procedure involved.

## Results

### The study sample

In all 400 women were studied. Of these, 218 women (54.5%) experienced a traumatic delivery. The mean age of participants (n = 218) was 26.9 (SD = 4.83) years. The mean number of children was 1.77 (SD = 0.86), and 119 women (54.6%) were experiencing their first childbirth. Thirty-four women (15.6%) completed their formal education at higher level. Forty-nine women (22.5%) had unwanted pregnancy; and 15 women (6.9%) had less than 8 times prenatal care visits (lower than the routine care). There were 139 women (63.8%) who had complicated pregnancy and 13 women (6.0%) had hospital admissions due to these complications. The duration of labor was lower than 3 hours in 32 women (14.7%), 67 women (30.7%) had emergency cesarean section, 60 (27.5%) had elective cesarean section, and 91 (41.7%) had normal vaginal delivery. Eighteen women (8.3%) had preterm deliveries and 14 women (6.4%) have delivered low birth weight babies (<2.5 kg). Overall, 80 women (20%) met the criteria for post-partum PTSD. Further analysis of the data showed that 131 women (60.1%) were suffering from re-experiencing, 91 (41.7%) from avoidance, and 120 (55.0%) from increased arousal. The detailed results are shown in Table 1.

**Table 1:** The characteristics of study sample

	**Traumatic birth (n = 218)**	**With PTSD (n = 80)**	**Without PTSD (n = 138)**	
		**No (%)**	**No (%)**	**P**
**Age groups (years)**				0.80
<20	19 (8.7)	8 (10.0)	11 (8.0)	
21-36	189 (86.7)	69 (86.2)	120 (87.0)	
>36	10 (4.6)	3 (3.8)	7 (5.1)	
**Education**				0.003
Primary	85 (39)	38 (47.5)	47 (34.1)	
Secondary	99 (45.4)	38 (47.5)	61 (44.2)	
Higher	34 (15.6)	4 (5.0)	30 (21.7)	
**Employment status**				0.152
House wife	196 (89.9)	5 (6.2)	17 (12.3)	
Employed	22 (10.1)	75 (93.8)	121 (87.7)	
**Number of children**				0.707
>1	99 (45.4)	35 (43.8)	64 (46.4)	
1	119 (54.6)	45 (56.2)	74 (53.6)	
**Pregnancy was planned**				0.007
Yes	169 (77.5)	54 (67.5)	115 (83.3)	
No	49 (22.5)	26 (32.5)	23 (16.7)	
**Gestational age at delivery**				0.001
Term	200 (91.7)	67 (83.8)	133 (96.4)	
Preterm	18 (8.3)	13 (16.2)	5 (3.6)	
**Number of prenatal care visits**				0.004
>10	163 (74.8)	52 (65.0)	111 (80.4)	
8-10	40 (18.3)	17 (21.2)	23 (16.7)	
<8	15 (6.9)	11 (13.8)	4 (2.9)	
**Complications due to pregnancy**				<0.001
Yes	79 (36.2)	41 (51.2)	38 (27.5)	
No	139 (63.8)	39 (48.8)	100 (72.5)	
**Admission due to pregnancy complications**				0.012
Yes	13 (6.0)	9 (11.2)	4 (2.9)	
No	205 (94.0)	71 (88.8)	134 (97.1)	
**Pregnancy intervals (year)**				<0.001
>2	194 (89.0)	61 (76.2)	133 (96.4)	
<2	24 (11.0)	19 (23.8)	5 (3.6)	
**Labor duration in hours**				0.007
Cesarean section	127 (58.3)	54 (67.5)	73 (52.9)	
>9	20 (9.2)	10 (12.5)	10 (7.2)	
3-9	39 (17.9)	12 (15.0)	27 (19.6)	
<3	32 (14.7)	4 (5.0)	28 (20.3)	
**Mode of delivery**				0.006
SVD	91 (41.7)	26 (37.5)	65 (47.1)	
ELCS	60 (27.5)	19 (23.8)	41 (29.7)	
EMCS	67 (30.7)	35 (43.8)	32 (23.2)	
**Admission due to post-partum complications**				0.10
No	204 (93.6)	72 (90.0)	132 (95.7)	
Yes	14 (6.4)	8 (10.0)	6 (4.3)	
**Sex of neonate**				0.315
Male	116 (53.2)	39 (48.8)	77 (55.8)	
Female	102 (46.8)	41 (51.2)	61 (44.2)	
**Birth weight (gr.)**				0.068
<2500	14 (6.4)	2 (2.5)	12 (8.7)	
2500-4000	194 (89.0)	72 (90.0)	122 (88.4)	
>4000	10 (4.6)	6 (7.5)	4 (2.9)	
**History of child morbidity**				0.009
Yes	64 (29.4)	32 (40.0)	32 (23.2)	
No	154 (70.6)	48 (60.0)	106 (76.8)	

### Risk factors for post-partum PTSD

The relationship between post-partum PTSD and independent variables was examined by performing forward conditional multiple logistic regression analysis. The diagnosis of post-partum PTSD was considered as outcome variable and all significant findings in univariate analyses were considered as independent factors. As shown in Table 2 the results indicated that education (OR for higher education = 0.15,%95 CI = 0.04-0.58, P = 0.006), gestational age (OR for prematurity = 6.43,%95 CI = 1.78-23.24, P = 0.004), number of prenatal care visits (OR for less than 8 times visits = 5.70,%95 CI = 1.40-23.24, P = 0.01), pregnancy complications (OR = 2.57,%95 CI = 1.26-5.22, P = 0.009), pregnancy intervals (OR for less than 2 years = 9.08,%95 CI = 2.75-29.97, P < 0.001), duration of labor (OR for less than 3 hours = 0.11,%95 CI = 0.03-0.45, P = 0.002), and mode of delivery (OR for emergency cesarean section = 4.88,%95 CI = 2.06-11.58, P < 0.001) were significant contributing factors to post-traumatic stress disorder after childbirth.

**Table 2:** The results obtained from multiple logistic regression analysis indicating risk factors for post-partum PTSD (n = 218)

	**OR (95% CI)**	**P**
**Education**		
Primary	1.0 (ref.)	
Secondary	0.56 (0.26-1.18)	0.13
Higher	0.15 (0.04-0.58)	0.006
**Gestational age at delivery**		
Term	1.0 (ref.)	
Preterm	6.43 (1.78-23.24)	0.004
**Number of prenatal care visits**		
>10	1.0 (ref.)	
8-10	1.45 (0.59-3.59)	0.41
<8	5.70 (1.40-23.24)	0.01
**Complications due to pregnancy**		
No	1.0 (ref.)	
Yes	2.57 (1.26-5.22)	0.009
**Pregnancy intervals**		
>2	1.0 (ref.)	
<2	9.08 (2.75-29.97)	< 0.001
**Labor duration in hours**		
Cesarean section	1.0 (ref.)	
>9	0.38 (0.09-1.45)	0.10
3-9	0.34 (0.12-0.91)	0.03
<3	0.11 (0.03-0.45)	0.002
**Mode of delivery**		
SVD	1.0 (ref.)	
ELCS	0.98 (0.39-2.24)	0.96
EMCS	4.88 (2.06-11.58)	< 0.001

## Discussion

The findings from present study indicated that more than half of women experienced a traumatic delivery. In addition we found that post-partum PTSD was associated with low educational level, premature labor, inadequate prenatal care visits, having complications due to pregnancy, pregnancy intervals less than 2 years, labor duration, and emergency cesarean section.

There are many studies that investigated about post-partum PTSD [3,26-29]. For instance a study from Australia reported that out of 499 women 5.6% met the DSM-IV criteria for post-partum PTSD 4–6 weeks after birth [3]. Prevalence of post-traumatic stress symptoms after childbirth reported to be varying from 1.5% to 32.1% [26,27]. However, this study found that 80 women (20%) were suffering from post-partum PTSD. This high prevalence of post-partum PTSD might be explained by the fact that we assessed post-partum PTSD early after delivery. There is evidence that the prevalence of post-partum PTSD usually is high when assessed early after childbirth [3]. Other reasons for this high prevalence of post-partum PTSD might include: the instrument used to measure PTSD, the sample characteristics [5], prevalence of traumatic birth experiences, the way that health care professionals within hospitals treated the women, and finally the high rate of emergency cesarean section.

It has been shown that emergency cesarean section compared to elective cesarean section and normal vaginal delivery was more unpleasant birth experience and also was a risk factor for development of post-partum PTSD [27,30]. In fact is argued that emergency cesarean section can be an obstetric predictor of the development of post-partum PTSD [3,16,30]. In addition it has been shown that the emergency cesarean section was a risk factor for post-partum mental health problems [31]. There is evidence that women who undergo an emergency cesarean section have most negative cognitions and emotional feelings regarding delivery [32].

Unlike the study by Adewuya et al. that did not find any association between socio-demographic factors and post-partum PTSD [16], the findings of current study showed that level of education was a significant contributing factor to the outcome. In fact the results obtained from this study indicated that women with lower educational level were more likely to present with post-partum PTSD. The probability of reduced risk factor for post-partum PTSD in women with higher educational level compared to those with lower level higher was very meaningful (OR 0.15 vs. 1.0 respectively as shown in Table 2). Perhaps this finding is a pointer to the issue of educational inequalities that impacts women’s health and as suggested at first instance it should be recognized and then be eliminated and if not at least be reduced [33].

Duration of labor and complications of pregnancy were found to be associated with post-partum PTSD. Studies showed that the increased medical interventions during labor, more intense pain in the first stage of labor and prolonged labor duration and perinatal complications similarly increase the likelihood of unfavorable birth experiences and consequently resulting in developing post-traumatic stress symptoms [27,34].

The finding from this study showed that preterm labor was associated with post-partum PTSD. Similarly, studies have reported that the birth of a premature infant was a predictor for the development of post-traumatic stress symptoms [35].

Prenatal care is an essential reproductive health service, and demonstrated that women who regularly see their health care providers during their pregnancy are less likely to expereince serious pregnancy complications [36]. The findings from present study also indicated that the number of prenatal care visits was associated with decreased post-partum PTSD. Perhaps women with increased prenatal care were more likely to receive additional support from health care providers and thus showed less post-partum PTSD. A study reported that reducing the number of antenatal care visits in low-risk pregnancies increases perinatal mortality in low- and middle-income countries [37]. It is argued that antenatal care is a preventive public health intervention designed to promote mother and infant health. However, Adewuya et al. found factors that independently associated with post-partum PTSD included hospital admission due to pregnancy complications, instrumental delivery, and emergency cesarean section, manual removal of placenta and poor maternal experience of control during childbirth [16]. Similarly, Soet et al. suggested that obstetric interventions may play a role in perceiving childbirth as traumatic, but other more personal or subjective factors have a mediating role in the subsequent development of post-traumatic stress symptoms [34].

Although unwanted pregnancy was significant factor in univariate analysis (see Table 1), its effect was diminished in the presence of other factors when performing forward conditional regression analysis. However, it has been suggested that unwanted pregnancy might be due to low-level health literacy, inappropriate contraception use, predominant male power, and even experience of traumatic events such as sexual abuse, or rape. As the result, it is obvious that in such situations post-partum PTSD would be expected and this is why we think empowering women should be a priority for public health interventions. Unfortunately we did not collect data on these and it would be helpful to consider such information in future studies.

In general, both fathers and mothers might be affected by a traumatic childbirth, but mothers are more vulnerable to PTSD, especially those who are unprivileged. Unfortunately in developing countries, there are few supporting services for women who exposed to a traumatic childbirth [38], although in some Muslim countries such as Iran family-based supports exist and women, to a large extent, are relying on such a support system and not official supporting services.

### Limitations

This study had some limitations. Firstly, this was a cross-sectional study and thus the results should be interpreted with caution. Secondly, we only included demographic and obstetric and perinatal factors in the analysis, while post-partum PTSD symptoms may occur among women who have experienced a history of trauma regarding other factors including locus of control, perceptions of the mother-child relationship, partner attachment and perception of partner support. Also we obtained our sample from public health services, thereby excluding women who did not use any healthcare facilities. Finally, since women from different cultural backgrounds might show different patterns for post-partum PTSD, the results from this study could not be generalized to western women or even all women from Iran.

## Conclusion

The findings indicated that the prevalence of traumatic birth experiences and post-partum PTSD were relatively high among Iranian women. The findings also indicated that obstetric and perinatal variables were independently the most significant contributing factors to women’s post-partum PTSD. It seems that a better care, supportive childbirth, and attention paid to human rights for women might help to reduce the burden of post-partum PTSD among this population.

## Competing interests

The authors declare that they have no competing interests.

## Authors' contributions

MM supervised the study. SA was the main investigator, designed the study, and collected the data. PR helped the main investigator to analyze the data and wrote the first draft. AM contributed to the analysis, critically evaluated the paper, and provided the final draft. All authors read and approved the final revision of the manuscript.

## Pre-publication history

The pre-publication history for this paper can be accessed here:

http://www.biomedcentral.com/1471-2393/12/88/prepub
